# Asylums for Idiots

**Published:** 1858-01

**Authors:** 


### Asylums for Idiots.

We perceive from a statement in the Boston Medical Journal that on Wednesday the 9th of December an interesting meeting was held in Philadelphia, upon the subject of Asylums for Idiots, by the Representatives of the following Institutions: "The Massachussetts School for Idiotic and Feeble minded youth," at Boston; "The Massachussetts Private Institution for Idiotic, Imbecile and Backward Children," at Barre, Massachussetts; "The New York Asylum for Idiots," at Syracuse; "The Ohio Asylum for Idiots," at Columbus; "The Pennsylvania Institution," at Germantown. The object of the meeting was to organize an association for the purpose of bringing the subject of Idiocy before the people, discussing questions of interest, and maturing methods of treatment, relative to this unfortunate class of human beings. As the result of these discussions, which were purely practical, certain principles were adopted. The next meeting of the Association will be held in New York. We hope before many years that the Legislature of Georgia will do something for the relief of this piteable portion of our fellow creatures, by the establishment of an Asylum specially designed and adapted to there condition. Under the present arrangement—placing them in the "Lunatic Asylum of the State"—that Institution is not only crowded with those for whose proper management their is no preparation, but it is prevented from giving accomodation to those for whose special benefit it was established.

With this change, and the exclusions of Epileptics—who ought to be placed in Hospitals—and the completion of the magnificent building now in the course of construction, and the other liberal arrangements provided for at the recent session of the Legislature, we shall soon have a provision for the Insane, worthy of the great State of Georgia.

